# Participation in Population Health Interventions by Older Adults in Poland: Barriers and Enablers

**DOI:** 10.3390/ijerph20032284

**Published:** 2023-01-27

**Authors:** Dorota Cianciara, Katarzyna Lewtak, Anna Poznańska, Maria Piotrowicz, Małgorzata Gajewska, Ewa Urban, Larysa Sugay, Anna Rutyna

**Affiliations:** 1 Department of Epidemiology and Health Promotion, School of Public Health, Centre of Postgraduate Medical Education, 01-826 Warsaw, Poland; 2 Department of Health Promotion and Prevention of Chronic Diseases, National Institute of Public Health NIH—National Research Institute, 00-791 Warsaw, Poland; 3 Department of Social Medicine and Public Health, Medical University of Warsaw, 3 Oczki Street, 02-007 Warsaw, Poland; 4 Department of Population Health Monitoring and Analysis, National Institute of Public Health NIH—National Research Institute, 00-791 Warsaw, Poland

**Keywords:** older adults, active ageing, health programmes, social activity, barriers and enablers

## Abstract

Background: The participation of older adults in population health interventions constitutes a key factor in their physical, mental and social health. The aim of this study was to determine variables considered as enablers and barriers to participation in health programmes. Methods: The conceptual framework of the study was developed and population health interventions were operationalised as health programmes. A total of 805 older adults participated in a questionnaire survey. The questionnaire included questions about socio-demographic, health and social connectedness-related factors as well as participation in population health interventions/programmes. Multiple logistic regression was used to examine the relationship between respondents’ characteristics and participation in the intervention. Results: Participation in health programmes was declared by 316 respondents. The enablers of participation were general practitioner’s affability (OR = 2.638 [1.453–4.791], *p* = 0.001), three or more social activities (OR = 3.415 [1.477–7.894], *p* = 0.004), taking part in support groups (OR = 4.743 [1.255–17.929], *p* = 0.022) and involvement in Universities of the Third Age (OR = 2.829 [1.093–7.327], *p* = 0.032). The barriers were primary education (OR = 0.385 [0.215–0.690], *p* = 0.001), infrequent general practitioner’s appointments (OR = 0.500 [0.281–0.888], *p* = 0.018) and lack of social activity (OR = 0.455 [0.299–0.632], *p* < 0.001). Conclusion: The enablers of participation appeared to solely include variables regarding health service utilisation, patient experience and social activity, i.e., interpersonal and community relationships, not intrapersonal factors.

## 1. Introduction

The phenomenon of ageing of populations has been observed both on a global scale and in individual countries. In 2018, the number of people aged 60 years or over in Poland was 9,392,700, which constituted 24.45% of all the country’s inhabitants [1]. In the same year, persons aged 60 years and more constituted 26.3% of the whole EU population [2]. According to the projection of Statistics Poland, the same population in 2030 will increase up to 10.8 million and 13.7 million in 2050. Then the group of older people will represent about 40% of total Polish population [3]. The consequences of this situation for health and social systems, their workforce and budgets are profound. According to the World Health Organization (WHO), the main challenge of healthcare systems is to promote health of older adults through activities focused on the variety of factors determining health. Involvement of older people is also justified on economic grounds, as it results in reduced expenditure on healthcare of this group while bringing economic benefits created by, inter alia, active participation of older people in the job market [4].

Population health interventions should be understood as approaches towards policies, programmes and resources that influence a number of people by modifying the distribution of health determinants to health improvement. As such, they have a significant potential for improving the health state on a population level [5]. Policy and programme interventions can operate within or outside of the health sector. The term ‘population health’ is used according to the Canadian standpoint and refers to a public health approach [6]. Population health interventions are considered as complex and context dependent; they should be theory driven and structured in the form of a network [7,8].

Population health interventions, which combine both clinical and community-based activities, seem to be of particular importance for older people since they address their health and social needs. In such instances, it is feasible to merge actions undertaken on the individual, interpersonal and social level, including health education, primary prevention, screening, rehabilitation, health promotion, support or the environmental changes in order to improve the functioning of older adults. The previous research abounds in examples of healthcare programmes addressed at older people which bring health-related benefits and are cost-effective [4,9]. The research evidence indicates that, from a global perspective, the number of interventions addressed at older adults has increased and they have become more diversified [10,11,12].

There is a proliferation of population health interventions observed in Poland, especially regarding health programmes. A health programme needs to respond to health needs and activate community resources. It needs to be founded on evidence-based justification as to the expected results, be well-planned, monitored and evaluated. In practice, in Poland, the aforesaid principles are sometimes omitted, which results, inter alia, from the diversity and superficiality of legal regulations on the health programmes’ implementation. As a result, health programmes assume various forms and names (e.g., programme, activity, action, campaign, championship, fair, festival, picnic, quiz competition). They are carried out by various operators, have varied goals, target groups, sources of funding, etc. Additionally, they differ in terms of monitoring and evaluation methods, due to the fact that the national public health reporting system does not take into consideration such key evaluation indicators as outcome and impact indicators.

With such a variety of health programmes, there is also an array of works addressed directly at older adults. For instance, one of the objectives of the 2016–2020 National Health Programme was to promote healthy and active ageing. In line with this objective, 1004, 1245, 1321, 1304 and 992 works were implemented in Poland in the individual years of this period (respectively). Wherein, the term ‘works’ should be understood similarly to the Norwegian Public Health Act [13] as efforts of primary and secondary prevention of mental and somatic illness, disorders and injuries, rehabilitation and health promotion. Nowadays in Poland, it is rather impossible to carry out a comprehensive and objective quality assessment and effectiveness evaluation for such diversified population health interventions addressed at older adults. However, random assessments are conducted, i.e., with regard to selected interventions, including those which refer to participants’ satisfaction [14,15]. Simultaneously, the conducted formative evaluations of the aforesaid interventions are insufficient, which impede the assessment of feasibility, appropriateness and acceptance before the implementation. In our study, we focused on certain factors that contribute to the participation of older people in population health interventions and that represent the demand side of services access [16]. 

The aim of the conducted study was to determine the socio-demographic factors and variables regarding health and social connectedness that demonstrate a positive (enablers) or negative (barriers) relationship with the participation of older adults in population health interventions.

## 2. Materials and Methods

### 2.1. Conceptual Framework 

In the context of this cross-sectional study, population health intervention was operationalised as a health programme, namely including the following:

- An undertaking (work);
- Organised by public healthcare and/or educational facilities, non-governmental organisations, enterprises or other entities;
- Promoted as the following:
  o Free-of-charge and available for specific groups of people (e.g., age, gender) within a particular period, beneficiaries inhabiting a particular place;
  o With the purpose to improve or maintain health.

**Figure 1:**
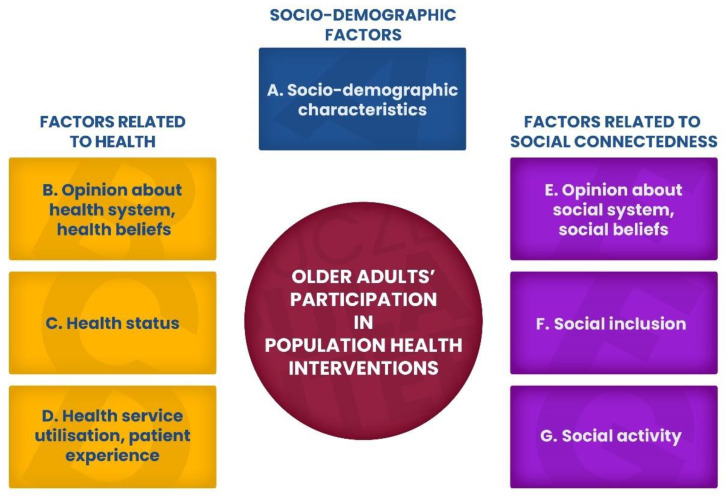
Conceptual framework of the study.

A conceptual framework that was created comprised seven constructs related to participation in population health interventions, hereinafter referred to as health programmes (Figure 1). It was assumed that construct A indicates socio-demographic variables, constructs B–D display factors related to health, while constructs E–G are factors related to social connectedness.

### 2.2. Methods

Between 11 and 17 December 2018, a CATI (computer assisted telephone interviewing) study was carried out on a quota sample of 805 residents of Poland aged 60+, with quotas for sex, age, size of the locality of residence and region based on the Eurostat classification, i.e., NUTS (Nomenclature of Territorial Units for Statistics) [17], according to Statistics Poland data [18]. Inclusion criteria were the age of 60 and above, knowledge of the Polish language and possession of a Polish telephone number. 

The questionnaire draft was developed by the researchers and was subsequently piloted twice. The research questionnaire was prepared in Polish and comprised 35 questions (34 close-ended and 1 open-ended) divided into three sections (socio-demographic factors—10 questions, factors related to health—15 questions, and factors related to social connectedness—10 questions). The final version of the instrument was handed over to the polling company. The procedure of drawing and conducting the telephone survey was carried out by this specialised polling company. Respondents were randomly selected from a database/registry of telephone numbers. The average duration of an interview was 25 min. The current analysis uses only data from close-ended questions.

During the interview, each respondent was presented with the adopted definition of a health programme with examples (e.g., immunisations, physical activities, informational and educational meetings) and asked about their own participation in such programmes. They also were asked to provide the information on their socio-demographic situation as well as social and health functioning. 

### 2.3. Statistical Analysis 

Based on the distribution of responses to the survey questions, the characteristics of the studied group (Table 1) and the frequency of participation in health programmes depending on particular features were found using descriptive statistics.

The statistical analysis was carried out in two steps. First, the odds ratio values (with the limits of 95% confidence intervals) of respondents with particular characteristics participating in the health programmes vs. people without these features were calculated. This way, the factors significantly related to participation were identified (Table 2). They were used as explanatory variables in the second step of the analysis, where the multiple logistic regression method (using Wald forward selection) was applied. It allowed us to eliminate the correlation between the explanatory variables and to assess the strength of association between the characteristics of respondents and their participation in health programmes (outcome variable). The significance level of 0.05 was assumed in the study. All statistical analyses were conducted using SPSS, version 27.0 (SPSS Inc., Chicago, IL, USA).

### 2.4. Material

The researched sample comprised 805 people aged 60 years and more, including 59% of women and 41% of men, which reflects the proportion of both sexes in this age group in the country’s population. The average age for members of the researched sample was 69.6 (median = 68); nearly 57% of the respondents were aged 60–69.

Detailed characteristics of the respondents are shown in Table 1. 

Considering the declaration of participation in health programmes, the respondents were divided into two groups: those who participated in programmes in the 6 month period preceding the survey and those who reported non-participation (i.e., 316 vs. 489 respondents). Ultimately, 39.3% of all respondents were included into the group benefiting from health programmes. Most of them participated in programmes aimed at the rehabilitation (19.0%), immunisation (12.0%) and health education (9.7%).

## 3. Results

### 3.1. Scope of Participation in Health Programmes

**Figure 2:**
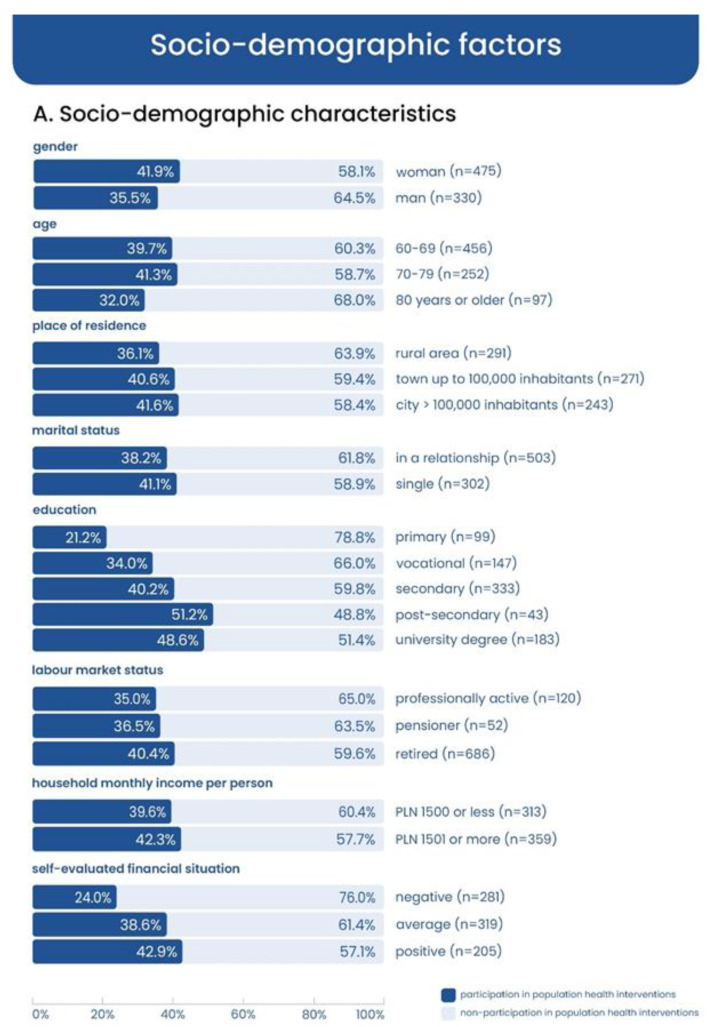
Participation in health programmes in subgroups (participating and non-participating) based on socio-demographic factors (in percentage).

**Figure 3:**
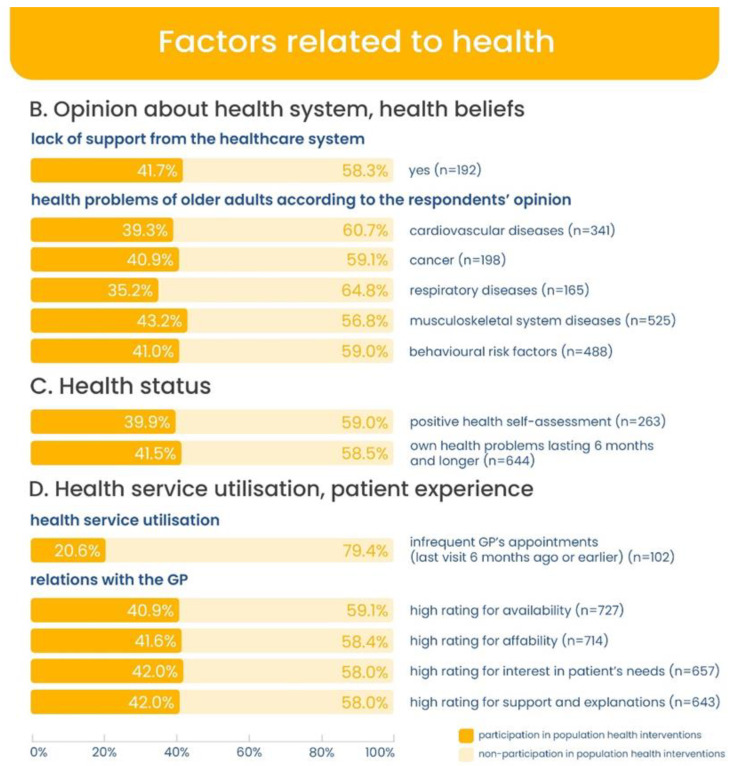
Participation in health programmes in subgroups (participating and non-participating) by health-related variables (in percentage).

**Figure 4:**
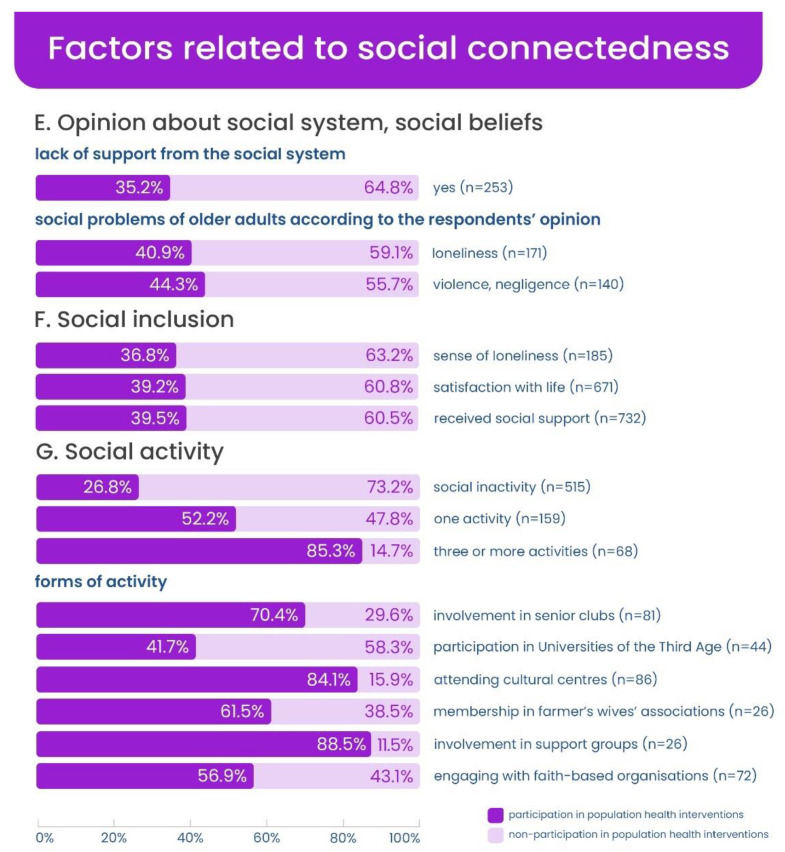
Participation in health programmes in subgroups (participating and non-participating) by social connectedness variables (in percentage).

The distribution of participation in health programmes depending on socio-demographic situation as well as variables related to health and social connectedness is shown in Figure 2, Figure 3 and Figure 4.

### 3.2. Enablers and Barriers to Older Adults’ Participation in Health Programmes 

Based on the data obtained through the interview, factors that could determine participation in health programmes were identified. Measures of strength of association (OR) and their statistical significance are presented in Table 2.

Not all groups of factors (A–G) shown in Figure 1 describing the respondents’ socio-demographic situation as well as health and social connectedness-related variables proved to be significant for the participation of older adults in health programmes.

The study showed that out of all the factors in group E (opinion on social system, social beliefs) and group F (social inclusion) none proved to be in a statistically significant relationship to participation in programmes. Significant relationships, on the other hand, were discovered in relation to some variables in group A (socio-demographic characteristics), B (opinion on health system, health beliefs), C (health status). These were (A) primary education (OR = 0.375 [0.226–0.621], *p* <0.001), university degree (OR = 1.648 [1.181–2.298], *p* = 0.003), negative self-evaluated financial situation (OR = 0.668 [0.493–0.904], *p* = 0.009), (B) perception of musculoskeletal system diseases as a health problem for older adults (OR = 1.619 [1.118–2.345], *p* = 0.011) and (C) own health problems lasting 6 months and longer (OR = 1.619 [1.118–2.345], *p* = 0.011). In group D (health service utilisation, patient experience) and group G (social activity), all variables proved to have a statistically significant relationship with participation in health programmes (Table 2). 

The factors significantly related to participation in health programmes are interrelated. In order to eliminate these dependencies, the multiple logistic regression method was used. Participation in population health interventions was considered a dependent variable. The explanatory variables were features indicated as significant in the univariate analysis.

**Figure 5:**
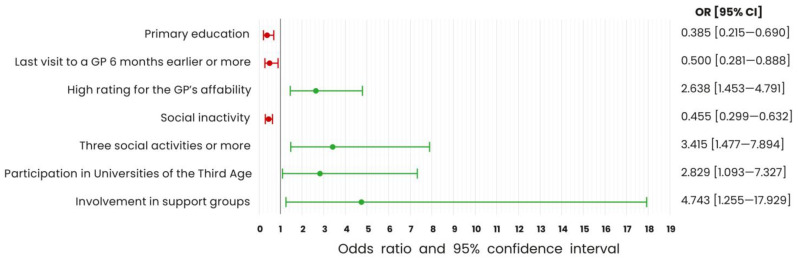
Characteristics significantly related with participation in health programmes (multiple logistic regression results).

The results of multiple logistic regression analysis are presented in Figure 5.

The enablers of participation were related to the following:

- Number of social activities: the chance of participation had increased more than threefold on the condition of participation in several forms of social activity. In the case of three or more social activities, the OR was 3.415 [1.477–7.894], (*p* = 0.004).
- Forms of social activities: engagement in support groups increased the chance of participation in health programmes as much as five times (OR = 4.743 [1.255–17.929], *p* = 0.022). A particularly important factor proved to be involvement in the University of the Third Age educational activities, which almost tripled the chance of using health programmes (OR = 2.829 [1.093–7.327], *p* = 0.032).
- Relation with the general practitioner (GP): his/her affability increased the chance of participation in population health interventions by 2.6 times (OR = 2.638 [1.453–4.791], *p* = 0.001).

The barriers were related to the following:

- Education: primary level (OR = 0.385 [0.215–0.690], *p* = 0.001) decreased the chance of participation by 61.5% in relation to people with higher education.
- Social activity: lack of social activity reduced the chance of participation by 54.5% (OR = 0.455 [0.299–0.632], *p* < 0.001).
- Health service utilisation: infrequent GP’s appointments halved the chance of participating (OR = 0.500 [0.281–0.888], *p* = 0.018).

**Figure 6:**
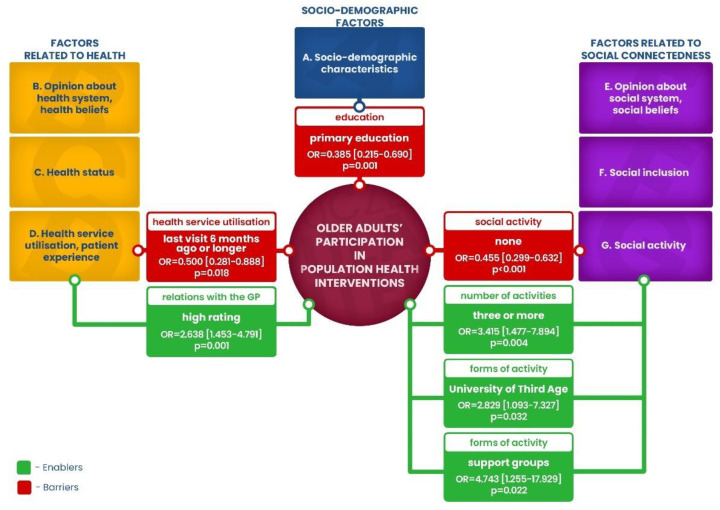
Barriers and enablers of participation for older people in health programmes.

The results of these analyses indicate that only some groups of factors were statistically significant in determining the participation of older adults in the population health interventions. Some variables classified as socio-demographic characteristics (A), those concerning health service utilisation and patient experience (D), as well as social activities (G) turned out to be statistically significant to participation in health programmes (Figure 6).

## 4. Discussion

The involvement of older adults in various forms of activity, including social, professional, cultural, educational or health activities, constitutes an important factor for improving their physical, mental, social health and wellbeing. Unfortunately, the idea and practice of active ageing are still a relatively new phenomenon in Polish circumstances. 

Involvement of older adults in activities promoting health is determined by many factors, including health condition, family and financial situation or level of education, which have a significant impact on their attitudes and choices [19]. The main focus of this study was to examine the extent of older people’s participation in population health interventions along with its determinants. Based on the literature, it was assumed that socio-demographic factors, similarly to variables concerning health situation, utilisation of healthcare services and social functioning play a crucial role regarding the involvement of older adults in health programmes [20,21].

In the conducted study, the term ‘health programme’ was used as the conversational illustration of population health interventions. The factors, which demonstrated the most significant association with participation in population health interventions (enablers) were high rating for the GP’s affability towards patients and the number and types of undertaken social activities. In both instances, the findings are in line with other studies. 

Publications on the involvement of the GP at various stages of the health programme implementation, inter alia through recommendation on the participation in a programme or participants’ support in the process of changes, proved that the GP has a crucial role in engaging patients in health interventions such as immunisation, lifestyle modification, regular medical check-ups or screening [22,23,24,25,26]. An important role in the implementation of health programmes is also played by the professional group of nurses, whose support takes the form of individual work with an older person or work in an interdisciplinary team [9,27]. 

The relationship between social activity and the use of various types of preventive health services has also been confirmed in numerous studies, e.g., in EU member states or Taiwan [28,29]. The study carried out in the UK by Stafford at el. [30], which analysed the involvement in various types of preventive health programmes including routine health check-ups, immunisation and cancer screening among older adults (68–69 years), might be considered as a further example. According to this study, people with poor social connectedness appear to be at greater risk of not engaging in the full range of preventive services compared to individuals with good social connectedness.

In the conducted study, factors decreasing odds of participation in health programmes (barriers) were relatively low level of education, rare contacts with a GP and social inactivity. There is an evident relationship between the socio-economic status, measured by a level of education, and the tendency to use various types of preventive services, which can be found in other studies. For instance, in Sweden and Norway, women with a low level of education are less likely to participate in cervical screening programmes [31,32]. In Denmark, primary education was indicated amidst the main predictors of non-participation in screening programmes among women by Kristensson et al. [33]. In Illinois, USA, in the study by Bobitt et al. [34], a lower degree of participation in Chronic Disease Self-Management Programmes and a lower completion ratio of the programme were identified among people with a lower level of education. However, results of the studies regarding the relationship between the level of education and immunisation are not always coherent. There are studies reporting a positive association between a high level of education and increased influenza vaccination, but at the same time other studies report a negative association, where a lower level of education resulted in higher rates of vaccination [35]. Additionally, there are studies proving that people with higher revenue and higher level of education exhibit higher acceptance for vaccination against COVID-19 [36,37]. 

The two other determinants of older adults’ non-participation in health interventions, i.e., rare visits to a GP or low social activity, are significantly important in Poland, where reports indicate low access to health services, negative opinions on doctors’ quality of work and a very low level of social activity among older people. According to the study carried out in Poland by the Public Opinion Research Center (CBOS) in August 2020, only half of the population had appointments with GPs in the 6 month period preceding the study, while in years 2012–2018, such services were utilised by three quarters of the population [38]. In 2021, as much as 30% of respondents of the national study, conducted by the same government agency, were clearly dissatisfied with the functioning of the healthcare system and 38% claimed that patients whose treatment was covered by public health insurance schemes experienced insufficient kindness and care. For comparison, in the 2007–2021 period, the percentage of people supporting the opinion that physicians treat their patients in a professional way, kindly and respectfully, decreased from 75% to 47% [39]. 

The social activity of older people in Poland is, in general, deficient, which is exemplified by levels of social isolation (10.6%) and involvement in non-religious nongovernmental organisations (6.3%) as well as affiliation to religious communities, organisations or groups (9.0%) [40]. The results from other studies also confirm that organisations for older adults are unpopular among Polish older people. The most favoured are the Universities of the Third Age. Before the onset of the COVID-19 pandemic, they were attended by 7.0% people aged 60 and over. Involvement in activities available at other organisations dedicated to older people (e.g., senior clubs, senior councils) was declared by only 1–5% [41].

One of the most popular forms of support provided to older adults in Poland are the above-mentioned Universities of the Third Age. Their main objectives are to conduct educational activities, integrate and activate older people in order to improve their quality of life and increase the participation in social life. In 2018, over 56% of Polish Universities of the Third Age functioned within the structure of non-governmental organisations, out of which 44.7% involved associations established solely for the purpose of running such an institution. In the 2017/2018 academic year, those universities involved 113,193 course students, mostly from Silesian Voivodeship (17,566), Masovian Voivodeship (14,997) and Greater Poland Voivodeship (11,410). The majority (86%) of the course students were people aged 61 and over [42].

The main document specifying the senior policy in Poland is the ‘Social Policy for Older Adults 2030. Safety—Engagement—Solidarity’. It replaced the previous document entitled ‘The presumptions of the long-term senior policy in Poland for the period of 2014–2020’. The current strategy is an appendix to the act of 11 September 2015 on older adults and, simultaneously, contributes to the vision of social and economic development of the country. As part of the senior policy, further complementary programmes are being implemented, i.e., the Social Activity of Older Adults for years 2014–2020, Senior+, CARE 75+ and Availability+ [43].

The government programme ‘Social Activity of Older Adults for years 2014–2020 (ASOS)’ involved financing of projects from four priority areas: (I) education, (II) social activity promoting intra- and intergenerational integration, (III) social participation and (IV) social services for older adults. In the 2016–2018 period, a total of 5221 bids were submitted, out of which 1127 projects were selected and co-financed by the government for the amount of PLN 113.5 million. The spectrum of those actions covered cultural and educational activities, such as health education, language courses, meetings with experts, one or several-day trips, workshops, as well as sport and artistic activities [43]. The ASOS programme was supported by the long-term programme called ‘Senior+’ (formerly the Senior Wigor programme) conducted by the Ministry of Family, Labour and Social Policy in the years 2015–2020. This programme intended to provide financial support for local governments, develop local support centres for older people and increase the number of participants in existing ‘Senior+’ entities [44].

Despite the previously mentioned attempts at activating older adults in Poland, their social activity is significantly lower than in other EU member states. According to the Active Ageing Index (AAI), elaborated on as part of the mutual project implemented by the European Commission and the United Nations Economic Commission for Europe (UNECE), the situation in Poland, compared to other EU member states, is unfavourable. The AAI includes 22 indicators grouped into four domains. According to the results of the AAI in 2018, the overall score ranks Poland 24th in the ranking of 28 EU member states. In the case of the score for the domain titled ‘participation in society’, Poland was ranked 23rd while in ‘capacity and enabling environment for active ageing’—21st [43]. The major reasons for the non-involvement of Polish older adults into the offer of senior organisations are limited free time resources, lack of identification with the senior organisations and different preferences regarding ways of spending free time or lack of awareness about the activities carried out by such organisations [44].

Since low social activity is associated with non-participation in health programmes, it should be considered as a significant stimulus prompting the formative evaluation and planning of population health intervention. There is a need for encouragement of participation in health programmes for socially inactive people, and providing them with a higher degree of services accessibility. Lack of efforts in this scope might raise or increase health inequities. According to reports, health inequities are connected with social network features. For instance, health inequities among people with lower socio-economic status are reduced through participation in social activities [45]. 

To summarise, there is a necessity to pay attention to the fact that participation in population health interventions was significantly associated with interpersonal factors (patient experience with GP, engagement in social activities) and were not associated with intrapersonal factors (e.g., opinion and beliefs about health and social systems and social issues affecting older people). Thus, it can be concluded that social factors are more important for older adults’ involvement in health programmes than individual factors. Consequently, external factors take priority over internal factors. As a consequence of such circumstances, it seems that the planning of population health interventions should be focused on interpersonal and community-level theories of behaviour change [46]. On the other hand, barriers were connected with individual factors (level of education), interpersonal relations (rare contact with a GP) and social relations (lack of social activity). It suggests that there is a more complex foundation for non-participation, which requires further in-depth studies.

Limitations of the work refer to the application of an exploratory rather than an explanatory approach, which resulted from the lack of prior research on the subject at the national level. Due to the fact that the study was based on self-reported data, its results can be biased because of, for instance, respondents’ selective memory or telescoping. Moreover, since the tool had to be adjusted to the time of interviews with older adults, the range of questions in the questionnaire was limited and some important factors connected with participation in population health interventions (e.g., functional dimensions of social networks such as quality of relationships) were omitted. 

## 5. Conclusions

The enablers of participation in population health interventions included variables concerning patient experience and social activity, i.e., high rating for the GP’s affability and considerable involvement in social activities. Thus, enablers were connected with interpersonal and community factors. The barriers included low level of education, relatively rare visits to a GP and non-participation in social activities. It means that barriers comprised mostly individual and interpersonal factors, which exemplifies the complex reasons behind non-participation. Older adults with a low level of education, seldom visiting their GP and with low social activity constitute a hard-to-reach group for population health interventions.

When planning and implementing interventions for older adults aimed at improving their health and reducing social health inequities, there is a necessity to take into consideration the current target group’s social activity along with recognizing specific enablers and barriers to participation. It involves the need for conducting formative evaluations and determining the external and internal factors related to participation. Further research in this field is required among the population of older adults in Poland, especially in the context of barriers to participation in population health interventions.

## Figures and Tables

**Table 1:** The frequency of occurrence for respondents’ selected characteristics.

Characteristcs		*n*	% of Total
**Socio-Demographic Factors**			
**A. Socio-Demographic Characteristics**			
Gender	Woman	475	59.0
	Man	330	41.0
Age	60–69	456	56.6
	70–79	252	31.3
	80 years or older	97	12.1
Place of residence	Rural area	291	36.1
	Town up to 100,000 inhabitants	271	33.7
	City >100,000 inhabitants	243	30.2
Marital status	In a relationship	503	62.5
	Single	302	37.5
Education	Primary	99	12.3
	Vocational	147	18.3
	Secondary	333	41.4
	Post-secondary	43	5.3
	University degree	183	22.7
Labour market status *	Professionally active	120	14.9
	Pensioner	52	6.5
	Retired	686	85.2
Household monthly income per person	PLN 1500 or less **	313	38.9
	PLN 1501 or more **	359	44.6
Self-evaluated financial situation	Negative	281	34.9
	Average	319	39.6
	Positive	205	25.5
**Factors** **Related** **to Health**			
**B. Opinion about Health System, Health Beliefs**			
Lack of support from the healthcare system		192	23.9
Health problems of older adults according to the respondents’ opinion	Cardiovascular diseases	341	42.4
	Cancer	198	24.6
	Respiratory diseases	165	20.5
	Musculoskeletal system diseases	525	65.2
	Behavioural risk factors	488	60.6
**C. Health Status**			
Positive health self-assessment		263	32.7
Duration of health problems	Lasting 6 months and more	644	80.0
**D. Health Service Utilisation, Patient Experience**			
Health service utilisation	Infrequent general practitioner’s (gp’s) appointments (last visit 6 months ago or earlier)	102	12.7
Relations with the general practitioner	High rating for availability	727	90.3
	High rating for affability	714	88.7
	High rating for interest in patient’s needs	657	81.6
	High rating for support and explanations	643	79.9
**Factors Related to Social Connectedness**			
**E. Opinion about Social System, Social Beliefs**			
Lack of support from the social system		253	31.4
Social problems of older adults according to the respondents’ opinion	Loneliness	171	21.2
	Violence, negligence	140	17.4
**F. Social Inclusion**			
Sense of loneliness		185	23.0
Satisfaction with life		671	83.4
Received social support		732	90.9
**G. Social Activity**			
Social inactivity		515	64.0
Number of activities	One	159	19.8
	Three or more	68	8.4
Forms of activity *	Involvement in senior clubs	81	10.1
	Participation in Universities of the Third Age	44	5.5
	Attending cultural centres	86	10.7
	Membership in farmer’s wives’ associations	26	3.2
	Involvement in support groups	26	3.2
	Engaging with faith-based organisations	72	8.9

* percentage values do not sum up to 100% as multiple answers were possible ** PLN 1500 = USD 316.3 based on the average exchange rate in 2022 according to the National Bank of Poland (Official Journal of NBP of 2022, item 10).

**Table 2:** Characteristics of respondents vs. participation in health programmes*.

Variable	Odds Ratio for Participation in Health Programmes (OR)	95% Confidence Interval Limits for OR	Significance (*p*-Value)
**Socio-Demographic Factors**			
**A. Socio-Demographic Characteristics**			
Woman	1.313	0.982–1.754	NS
Age of 60–69	1.043	0.784–1.388	NS
Age of 80 and more	0.697	0.443–1.096	NS
Place of residence—rural area	0.811	0.602–1.091	NS
Place of residence—city >100,000 inhabitants	1.148	0.845–1.560	NS
Marital status—in a relationship	0.886	0.662–1.186	NS
Primary education	0.375	0.226–0.621	<0.001
University degree	1.648	1.181–2.298	0.003
Professionally active	0.808	0.539–1.211	NS
Retired	1.389	0.920–2.098	NS
Household monthly income per person below PLN 1500	1.025	0.767–1.370	NS
Negative self-evaluated financial situation	0.668	0.493–0.904	0.009
**Factors** **Related** **to Health**			
**B. Opinion about Health System, Health Beliefs**			
Lack of support from the healthcare system	1.141	0.820–1.587	NS
Cardiovascular disease as a health problem of the population of older adults	1.003	0.753–1.335	NS
Cancer as a health problem	1.096	0.790–1.520	NS
Respiratory diseases as a health problem	0.803	0.562–1.146	NS
Musculoskeletal system diseases as a health problem	1.635	1.205–2.218	0.002
Behavioural risk factors as a health problem	1.203	0.899–1.610	NS
**C. Health Status**			
Positive self-assessment of health	1.043	0.771–1.409	NS
Own health problems lasting 6 months and longer	1.619	1.118–2.345	0.011
**D. Health Service Utilisation, Patient Experience**			
Visit to a GP 6 months earlier or more	0.359	0.217–0.593	<0.001
High rating for GP’s availability	2.145	1.253–3.672	0.005
High rating for GP’s affability	2.699	1.593–4.571	<0.001
High rating for GP’s interest in patient’s needs	1.956	1.318–2.902	<0.001
High rating for GP’s support, Explanations	1.825	1.254–2.658	0.002
**Factors Related to Social Connectedness**			
**E. Opinion about Social System, Social Beliefs**			
Lack of support from the Social system	0.707	0.571–1.058	NS
Loneliness as a social problem	1.093	0.775–1.542	NS
Violence, negligence as a social problem	1.286	0.890–1.859	NS
**F. Social Inclusion**			
Sense of loneliness	0.872	0.621–1.224	NS
Satisfaction with life	0.985	0.674–1.440	NS
Received social support	1.111	0.676–1.828	NS
**G. Social Activity**			
Social inactivity	0.230	0.169–0.313	<0.001
One social activity	1.936	1.364–2.747	<0.001
Three or more social activities	10.768	5.412–21.425	<0.001
Involvement in senior clubs	4.264	2.585–7.034	<0.001
Participation in Universities of the Third Age	9.132	4.017–20.758	<0.001
Attending cultural centres	6.656	3.910–11.332	<0.001
Membership in farmer’s wives’ associations	2.555	1.144–5.703	0.022
Involvement in support groups	12.717	3.785–42.723	<0.001
Engaging with faith-based Organisations	2.203	1.350–3.595	0.002

* odds ratio calculated in relation to respondents without the analysed feature. NS—*p* ≥ 0.05.

## Data Availability

The data that support the findings of this study are available from the corresponding author, upon reasonable request.
